# Exercise, Atherosclerosis, and Cardiovascular Outcomes: Evidence and Challenges

**DOI:** 10.3390/jcdd13100479

**Published:** 2026-09-23

**Authors:** Mohammadmoein Dehesh, Reihanehsadat Hosseini, Rias Ali, Jasleen Kaur, Todd C. Villines

**Affiliations:** 1 Department of Medicine, University of Central Florida, Pensacola, FL 32514, USA; mohammadmoein.dehesh@ucf.edu (M.D.); jasleen.kaur@hcahealthcare.com (J.K.); 2 Ciccarone Center for the Prevention of Cardiovascular Disease, Johns Hopkins University, Baltimore, MD 21287, USA; 3 College of Nursing, Walden University, Minneapolis, MN 55401, USA; reihanehsadat.hosseini@waldenu.edu; 4 Division of Cardiovascular Medicine, University of South Florida, Tampa, FL 33620, USA; rias.ali@hcahealthcare.com; 5 Division of Cardiovascular Medicine, University of Virginia Health System, Charlottesville, VA 22908, USA

**Keywords:** atherosclerosis, ASCVD, exercise, physical activity

## Abstract

Exercise is a cornerstone of cardiovascular primary prevention, yet its relationship with atherosclerosis is more complex than traditionally appreciated. Exercise improves the lipid profile by lowering total cholesterol, LDL-C, triglycerides, and apolipoprotein B, while increasing HDL-C and apolipoprotein A-I. It also enhances insulin sensitivity, lowers blood pressure, reduces body adiposity, and favorably influences glucose metabolism. In addition, it regulates oxidative stress and systemic inflammation, two key drivers of atherosclerosis. Exercise further exerts direct vascular effects by improving endothelial function, slowing plaque progression, and promoting a more stable plaque phenotype. Current evidence supports approximately 1000 MET-minute/week, roughly equivalent to 3 h/week of moderate-intensity exercise, as a pragmatic population-level target, with additional benefit from resistance training; however, the optimal prescription may vary among individuals. High-intensity interval training may further improve cardiorespiratory fitness and selected cardiometabolic parameters in appropriate individuals. Although highly trained endurance athletes may exhibit greater coronary artery plaque burden, available data suggest that their overall cardiovascular risk remains lower than sedentary individuals. In this review, we discuss the mechanisms through which exercise influences atherogenesis, review the evidence regarding different exercise types and patterns, and examine the clinical implications and future directions of exercise-based prevention strategies.

## 1. Introduction

Cardiovascular disease (CVD) is the leading cause of mortality worldwide and accounts for approximately 30% of annual deaths, with atherosclerosis representing the most common underlying cause [1]. Beyond mortality, CVD also imposes a substantial burden of disability and healthcare costs, making prevention a major global priority [2]. The global burden of cardiovascular diseases has been estimated at approximately 437 million disability-adjusted life years (DALYs) [3]. One explanation for this persistent burden may be the historical emphasis on treatment rather than prevention, particularly in the context of rising cardiometabolic risk factors such as sedentary lifestyle, obesity, and diabetes [4,5].

Current guidelines strongly advocate exercise and physical activity as key strategies for both primary and secondary prevention of atherosclerotic cardiovascular disease (ASCVD) [6,7], particularly given increasingly sedentary lifestyles [8,9].

Exercise is believed to improve vascular health through favorable effects on traditional risk factors and reduce the risk of cardiovascular events [10,11]. A meta-analysis of 37 cohorts demonstrated a strong inverse relationship between cardiorespiratory fitness (CRF) and all-cause mortality risk [12]. At the same time, some studies have found that high-intensity or long-term endurance exercise is associated with increased coronary artery calcification, although the clinical significance remains uncertain [11,13,14,15]. In addition, findings regarding the optimal volume, type, and intensity of exercise remain inconsistent [11]. The complicated and sometimes contradictory relationship between this key modifiable risk factor for CVD reveals a crucial knowledge gap: how can physical activity be optimized to maximize cardiovascular benefit while minimizing potential risks?

In this review, we explore the pathophysiology of atherosclerosis and the biological link between atherosclerosis and exercise. Then we summarize evidence from large clinical studies, most updated guidelines and address clinical implications for primary prevention.

## 2. Pathophysiology of Atherosclerosis

The endothelium is the cellular layer that separates circulating blood from the underlying tissues. Endothelial dysfunction is considered the earliest step in atherosclerosis [16,17]. Hemodynamic forces are among the main local contributors to this dysfunction. Atherosclerosis preferentially develops in arterial regions exposed to disturbed flow and low or oscillatory wall shear stress, such as bifurcations and branch points [16,18,19,20]. Loss of shear-stress-mediated nitric oxide (NO) signaling further promotes vascular injury [21,22]. In parallel, oxidative stress and chronic low-grade inflammation contribute to endothelial dysfunction and accelerate atherogenesis [23,24].

Infiltration of low-density lipoprotein (LDL) particles into the intima initiates atherosclerotic plaque formation. These particles enter the vessel wall through several mechanisms, including passive diffusion and vesicular transport [25,26,27]. Endothelial activation then leads to the expression of adhesion molecules such as vascular cell adhesion molecule 1 (VCAM-1), intercellular adhesion molecule 1 (ICAM-1), and selectins, which recruit circulating monocytes and T cells. Within the intima, monocytes differentiate into macrophages that engulf modified LDL, forming foam cells and, ultimately, fatty streaks [28,29,30]. In this setting, inflammation, which is normally protective, becomes chronic and maladaptive, and drives plaque progression.

Mural cells also have important and context-dependent roles in vascular disease. Vascular smooth muscle cells (VSMCs) migrate from the media into the intima, proliferate, and undergo phenotypic switching during atherogenesis. Collagen- and extracellular matrix-producing VSMCs contribute to fibrous-cap formation and plaque stability, whereas VSMC-derived foam cells, osteogenic transformation, senescence, and apoptosis may promote lipid accumulation, inflammation, calcification, and plaque vulnerability [31,32,33]. Within the microcirculation, pericytes surround endothelial cells and help regulate capillary stability, permeability, vascular tone, and angiogenesis. Pericyte dysfunction or loss may destabilize microvessels and contribute to impaired perfusion, inflammation, and intraplaque neovascularization [34].

As plaques evolve, they undergo positive remodeling, with outward arterial expansion that preserves lumen size and keeps the disease clinically silent in its early stages. With continued lipid retention and immune activation, lesions develop necrotic cores and fibrous caps, together with angiogenesis and intraplaque neovascularization. These fragile microvessels may rupture, contributing to intraplaque hemorrhage, fibrosis, and calcification [16,31,32,35].

Plaque stability depends largely on the thickness and integrity of the fibrous cap. Plaques with thin caps are more vulnerable to rupture or erosion [36,37]. Inflammation weakens the cap by reduction in collagen synthesis and extracellular matrix integrity [38,39,40,41]. Thrombus formation following plaque rupture or erosion is the key event leading to acute clinical manifestations such as myocardial infarction and stroke.

## 3. Exercise and Atherosclerosis: Biological Links

### 3.1. Lipid Metabolism

Exercise influences lipid metabolism by affecting several key enzymes involved in triglyceride clearance and lipoprotein remodeling. One of the most consistently observed adaptations is the upregulation of lipoprotein lipase (LPL) activity in skeletal muscle, but not in adipose tissue. LPL hydrolyzes triglyceride-rich lipoproteins such as chylomicrons and very-low-density lipoprotein (VLDL), releasing free fatty acids for oxidation. At the same time, exercise appears to reduce LPL activity in adipose tissue, thereby shifting lipid flux toward active skeletal muscle. This redistribution enhances postprandial triglyceride clearance and lowers circulating VLDL levels [42,43,44,45].

Exercise also affects enzymes involved in high-density lipoprotein (HDL) maturation and reverse cholesterol transport. Lecithin-cholesterol acyltransferase (LCAT), which esterifies free cholesterol on HDL particles and promotes the formation of mature HDL, is stimulated by physical activity. Higher LCAT activity is associated with higher high-density lipoprotein cholesterol (HDL-C) and apolipoprotein A-I (ApoA-I) concentrations [42,46].

Hepatic triglyceride lipase (HTGL) is another enzyme involved in lipid metabolism that is influenced by exercise. Exercise has been shown to reduce HTGL activity. Because HTGL lowers HDL levels and tends to produce smaller, less efficient HDL particles, its reduction is generally considered favorable. HTGL also contributes to the conversion of VLDL to LDL [42,47,48], including the generation of smaller, denser LDL particles that are more atherogenic [49].

Although individual studies have sometimes produced conflicting results, exercise-induced changes in lipid metabolism generally lead to lower plasma cholesterol, low-density lipoprotein cholesterol (LDL-C), and triglyceride levels, along with higher HDL-C and improved HDL-to-LDL ratios [42,50,51,52,53,54]. A meta-analysis of 148 randomized controlled trials published in 2025 showed that exercise training improved all five major lipid outcomes [55]. These anti-atherogenic effects have been observed not only in healthy individuals but also in patients with ischemic heart disease, diabetes, and renal failure [56,57]. Exercise has also been shown to reduce apolipoprotein B (ApoB) and increase ApoA-I, both of which are important predictors of coronary heart disease (CHD) risk [42,57,58].

### 3.2. Systemic Metabolic Effects

Regular physical activity is associated with broad metabolic benefits, particularly by improving insulin sensitivity [59,60]. Exercise increases glucose uptake in skeletal muscle through both insulin-dependent and insulin-independent pathways, largely via translocation of the GLUT4 transporter [61]. It also enhances insulin signaling by reducing intramyocellular lipid content, improving mitochondrial efficiency, and lowering systemic inflammation. These adaptations reduce fasting insulin levels and improve whole-body glucose disposal [62,63,64,65].

Exercise also contributes to lower blood pressure, as shown in multiple review articles [66,67]. These effects are driven by improvements in vascular function and autonomic balance. Proposed mechanisms include enhanced endothelial nitric oxide bioavailability [68,69,70], improved flow-mediated dilation, reduced peripheral vascular resistance [71,72], and decreased sympathetic nervous system activity [73]. The chronic blood pressure-lowering effects of exercise training should be distinguished from blood pressure reactivity during an acute exercise bout. An exaggerated blood pressure response during exercise may reflect impaired endothelial vasodilator function and early vascular dysfunction [74]. Furthermore, higher blood pressure during submaximal exercise and delayed post-exercise blood pressure recovery have been associated with future hypertension and adverse cardiovascular outcomes, including among apparently healthy or asymptomatic individuals [75,76]. However, there is no evidence that exercise itself causes hypertension. This finding may reflect the unmasking of individuals with early or underlying hypertensive physiology that is already associated with elevated future cardiovascular risk.

Exercise also influences body weight, adiposity, and body mass index (BMI), although its greatest metabolic impact may result from changes in body composition rather than weight alone. Training increases energy expenditure, preserves or increases lean mass, and preferentially reduces visceral adipose tissue, which is strongly linked to cardiometabolic risk [77,78].

Cardiovascular and metabolic adaptations to exercise are not uniform across individuals. Biological sex may influence these responses through differences in hormonal status, body composition, substrate utilization, autonomic regulation, and vascular function. Consequently, men and women may differ in their blood pressure responses, cardiac remodeling, metabolic adaptations, and recovery following exercise, although substantial variability exists within each sex [79]. Age, menopausal status, baseline fitness, comorbidities, and exercise modality should therefore be considered when developing individualized exercise prescriptions rather than applying a uniform approach.

### 3.3. Oxidative Stress

Exercise influences redox homeostasis in a dose- and context-dependent manner. Although acute vigorous exercise can transiently increase reactive oxygen species production, regular training enhances endogenous antioxidant defenses, including superoxide dismutase and glutathione peroxidase [80,81,82,83]. These adaptations may reduce chronic oxidative stress; however, much of the mechanistic evidence derives from experimental and skeletal-muscle studies. Adam et al. studied patients with CAD and showed that exercise training reduced NAD(P)H oxidase expression and activity, decreased reactive oxygen species (ROS) generation in internal mammary artery samples, and improved endothelium-dependent vasodilation [84].

### 3.4. Inflammation

Regular physical activity exerts strong anti-inflammatory effects that counter the chronic low-grade inflammation characteristic of atherosclerosis. Exercise reduces circulating levels of pro-inflammatory cytokines such as tumor necrosis factor-α (TNF-α) and interleukin-1β (IL-1β), while increasing anti-inflammatory mediators such as interleukin-10 (IL-10) and adiponectin. These changes reflect both reduced activity of visceral adipose tissue, which is an important source of inflammatory cytokines, and favorable changes in immune cell profiles. Repeated exercise promotes a shift from pro-inflammatory M1 macrophages toward more reparative M2 phenotypes within vascular and adipose tissue, thereby reducing the inflammatory environment that contributes to endothelial dysfunction and plaque development [85,86,87]. Regular training also downregulates nuclear factor-κB (NF-κB), a central transcription factor that regulates cytokine expression and adhesion molecule production in endothelial cells and monocytes [87,88].

Human training studies have also shown reductions in high-sensitivity C-reactive protein (hs-CRP) with regular aerobic exercise, even in the absence of major weight loss. This suggests that exercise reduces inflammation partly independent of body fat [89,90,91].

### 3.5. Endothelium, Microcirculation and Plaque Dynamics

The endothelium is widely distributed throughout the vasculature and responds to numerous physiological signals, coordinating both intra- and inter-organ communication. It also plays an important role in mediating many of the metabolic effects of exercise. Exercise influences coronary and arterial plaque dynamics in ways that extend beyond traditional risk-factor modification. Most studies suggest that exercise helps prevent plaque formation, improves endothelial function, reduces arterial stiffness, and promotes angiogenesis [92,93,94,95,96,97,98,99].

Exercise also affects the microcirculation, which regulates tissue perfusion and oxygen delivery independently of epicardial plaque burden. Repeated increases in blood flow and shear stress enhance nitric oxide bioavailability, improve endothelium-dependent vasodilation, and promote functional and structural adaptation of resistance vessels and capillary networks [8]. In a small randomized study of patients with coronary artery disease (*n* = 19), four weeks of exercise training improved endothelial function in coronary resistance vessels and increased coronary flow reserve [100].

Studies examining the correlation of exercise and coronary or arterial plaque burden have reported inconsistent findings. Comparisons across studies are challenging because populations vary in baseline characteristics, comorbidities, methods of exercise assessment, and imaging techniques. Several clinical studies report that athletes have greater coronary artery calcification and greater total plaque burden than sedentary individuals [13,14,101]. Additional studies suggest that although athletes may have more plaques overall, these plaques tend to be more calcified and potentially more stable [13,14]. However, a few studies have reported that endurance athletes may have more plaques including mixed and noncalcified plaques as well as a greater proportion of unstable lesions [102]. One limitation of many studies was the selection of comparison groups. Notably, two studies using matched cases and controls based on the Framingham Risk Score (FRS) still reported a higher coronary artery calcium (CAC) burden in athletes [14,103].

Beyond coronary artery studies, one carotid ultrasound study showed that regular physical activity was associated with slower progression of plaque burden and, in some cases, partial regression [104]. Two additional imaging studies reported reductions in plaque lipid volume and necrotic core [105,106]. To our knowledge, current evidence does not show that regular exercise in non-athletes increases plaque burden compared with sedentary individuals.

Animal studies, in which naturally control confounding factors better, consistently show that exercise, especially when initiated at younger ages, reduces plaque burden and stenosis while promoting more stable plaque characteristics [107,108].

Several mechanisms may contribute to increased plaque calcification in highly active individuals, including repeated hemodynamic stress during intense exercise, reduced local inflammation, transient increases in parathyroid hormone, and lower magnesium levels reported in some athletes [85,87,109,110,111,112,113,114].

## 4. Exercise and ASCVD: Large Clinical Studies

The Framingham Heart Study (FHS) is one of the oldest cardiovascular cohorts and began in 1948 with 4729 cardiovascular disease-free participants aged 30 to 60 years. A report in 2013 estimated the associations of physical activity with incident CVD and CVD-related mortality, with hazard ratios of 0.95 (95% CI 0.84–1.07) and 0.83 (95% CI 0.72–0.97), respectively. In that report, physical activity scores were based on self-reported daily hours of sleep, sedentary behavior, and slight, moderate, and heavy physical activity [115]. In a later FHS sub-study, 903 participants were evaluated objectively using smartwatch-derived physical activity data. A 2020 report showed that every additional 1000 steps of habitual physical activity was associated with a 0.18% lower CVD risk (*p* = 0.003) based on the 2013 American College of Cardiology/American Heart Association (ACC/AHA) pooled cohort equation [116].

The Atherosclerosis Risk in Communities (ARIC) study is another landmark cohort in the United States. ASCVD-free participants aged 45 to 64 years have been followed since 1989. In one analysis, 9996 individuals with self-reported physical activity data at visit 1 were followed for a mean of 20.9 years, during which 1723 ASCVD events were recorded. Moderate-to-vigorous physical activity, measured in minutes per week (min/w), was associated with a significantly lower incidence of ASCVD both in the presence and absence of a family history of premature coronary heart disease (CHD). The hazard ratio was 0.84 (95% CI 0.74–0.94) after adjustment for demographic and lifestyle factors [117]. Another report showed similar findings, with no significant difference between African American and White participants [118].

The Coronary Artery Risk Development in Young Adults (CARDIA) study followed 5115 participants aged 18 to 30 years at baseline in 1985–1986. Because this cohort began at a younger age than many other studies, it may provide a clearer temporal relationship between physical activity and later cardiovascular outcomes. Meeting the recommended minimum of 150 min/w of moderate-to-vigorous physical activity was inversely associated with both overall CVD (adjusted odds ratio [AOR] 0.74, 95% CI 0.57–0.96) and CHD (AOR 0.60, 95% CI 0.42–0.85) [119]. Another report from CARDIA showed that individuals who later developed CVD had consistently lower physical activity levels during the 12 years preceding the event, with a steeper decline in the final 2 years before the event. These individuals also maintained lower physical activity levels after the event [120].

The association between physical activity and cardiovascular outcomes has also been demonstrated across geographically and socioeconomically diverse populations. In the Prospective Urban Rural Epidemiology (PURE) study, which included 130,843 participants without CVD, from 17 countries, higher physical activity was associated with lower risks of mortality (HR 0.80, 95% CI 0.74–0.87, *p* < 0.0001) and major cardiovascular events (HR 0.86, 95% CI 0.78–0.93, *p* < 0.0001). Importantly, these associations were observed for both recreational and nonrecreational activity and across countries at different income levels [121].

The Multi-Ethnic Study of Atherosclerosis (MESA) is a multicenter US-based cohort that has followed 6777 participants aged 45 to 84 years since 2000–2001. In this cohort, among individuals with CAC < 100 Agatston units, the highest quartile of self-reported physical activity was associated with a lower risk of CVD compared with the lowest quartile. However, this association was not observed in participants with CAC ≥ 100 Agatston units. Importantly, high levels of physical activity were not associated with worse outcomes regardless of CAC category [122].

Population-based outcome studies should be considered alongside studies evaluating imaging-defined atherosclerosis. In CARDIA, cardiorespiratory fitness in young adulthood was inversely associated with developing CAC over 15 years (*p* = 0.03) [123]. In MESA, vigorous activity was modestly associated with a lower incidence of CAC (RR 0.97, 95% CI 0.94–1.00) [124]. However, other large cohorts have reported a positive association between high physical activity and CAC prevalence or progression. In a cohort of 25,485 adults free of CVD, undergoing serial CAC examinations, Sung et al. found a graded association between physical activity and CAC prevalence and progression regardless of baseline CAC scores, although the clinical significance of this finding remained uncertain [125].

More recent longitudinal studies have not consistently confirmed that high exercise volumes accelerate CAC progression. Among 8771 generally healthy adults with repeated CAC measurements over 7.3 year of follow-up, Shuval et al. found that high and very high physical activity levels were not associated with faster relative CAC progression or progression to a CAC score of 100 or greater [126]. In the Cooper Center Longitudinal Study, very high physical activity (≥3000 Metabolic Equivalent of Task-minute/week (MET-min/w)) was associated with a higher prevalence of CAC among generally healthy men but was not associated with increased cardiovascular or all-cause mortality, including among those with elevated CAC [127]. These findings underscore the importance of distinguishing an imaging marker of calcified plaque from subsequent clinical risk.

A large meta-analysis published in 2023 included data from 96 cohorts. The investigators analyzed CVD incidence and CVD mortality across 28,884,209 person-years, with a total of 74,757 events. Reported levels of physical activity were standardized into MET-hours/week (equal to 60 MET-min/w). In a curvilinear dose–response relationship, 525 MET-min/w was associated with a 27% lower risk of CVD incidence and a 29% lower risk of CVD-related mortality [128]. This amount of activity is roughly equivalent to the recommended 150 min/w of moderate-intensity activity, such as brisk walking [129]. Beyond approximately 600 to 900 MET-min/w, additional risk reduction was no longer statistically significant for either CVD incidence or mortality [128].

Table 1 summarizes major large-scale clinical studies. Collectively, large prospective cohorts consistently associate greater physical activity or cardiorespiratory fitness with lower cardiovascular morbidity and mortality. In contrast, studies using CAC or coronary computed tomography angiography (CCTA) demonstrate a more heterogeneous relationship between exercise and imaging-defined coronary atherosclerosis. Very high-volume or very vigorous endurance exercise, particularly among middle-aged and older men, may be associated with higher CAC scores or greater CCTA-defined plaque burden, but longitudinal findings remain inconsistent. Importantly, greater CAC among highly active individuals has not consistently translated into higher cardiovascular or all-cause mortality.

## 5. Exercise for Primary Prevention of ASCVD: Type, Duration, Intensity, Pattern

The 2019 ACC/AHA prevention guideline states that evidence remains insufficient to determine whether sedentary behavior independently worsens ASCVD outcomes [6]. The guideline supports aerobic exercise as a key strategy for ASCVD risk reduction and suggests an L-shaped dose–response relationship, meaning that the greatest benefit occurs when moving from inactivity to modest activity, with additional benefit at higher levels of exercise. A minimum of 150 min/w of moderate-intensity aerobic exercise or 75 min/w of vigorous-intensity aerobic exercise is recommended [6]. Also, the 2026 ACC/AHA lipid management guideline suggested ≥150 min/w moderate- to vigorous-intensity aerobic exercise along with upper- and lower-body resistance exercise 2 days/week in patients with dyslipidemia [131].

Despite extensive evidence supporting the role of exercise in ASCVD prevention, clinical studies directly comparing the effects of aerobic and resistance training on ASCVD outcomes remain limited. A recent review of resistance exercise in patients with coronary artery disease found that resistance training improves cardiovascular responses and cardiorespiratory fitness and that combined resistance and aerobic training may represent the most effective exercise strategy [132]. However, the 2019 ACC/AHA prevention guideline did not specifically quantify the effect of resistance exercise alone on ASCVD outcomes despite endorsing metabolic and functional benefits [6]. Conversely, the 2021 European Society of Cardiology (ESC) prevention guideline gave resistance exercise a class 1-B recommendation for ASCVD risk reduction, which is recommended with aerobic exercise [133]. A 2022 meta-analysis of seven cohorts involving 257,888 participants and 16,056 cardiovascular events found that muscle-strengthening activity was associated with a 17% lower risk of CVD (RR 0.83, 95% CI 0.73–0.93). The greatest benefit appeared at approximately 30–60 min/w, whereas the effects beyond 130–140 min/week remained uncertain [134]. The mechanisms underlying this J-shaped relationship remain uncertain, although increased arterial stiffness and inflammation have been proposed as possible contributors [135,136]. A 2025 meta-analysis of 148 randomized trials involving 8673 participants found modest improvements in total cholesterol (MD −5.90 mg/dL, 95% CI −8.14 to −3.65), HDL-C (+2.11 mg/dL, 95% CI 1.43–2.79), LDL-C (−7.22 mg/dL, 95% CI −9.08 to −5.35), and triglycerides (−8.01 mg/dL, 95% CI −10.45 to −5.58), with combined aerobic and resistance training considered the most effective approach [55].

Over the past several years, multiple systematic reviews and meta-analyses have studied exercise intensity and pattern, particularly high-intensity interval training (HIIT). Their findings have not been entirely consistent, in part because of differences in study populations, exercise evaluations, and outcome definitions. Some meta-analyses reported that HIIT was not superior to moderate-intensity continuous training for improving LDL-C or overall lipid profiles [137,138,139], whereas others found HIIT to be among the most effective modalities for lowering LDL-C [140,141,142]. A more recent and reportedly most comprehensive meta-analysis by Ding et al. assessed 49 clinical trials and included dose–response analyses. That study found that HIIT and combined aerobic plus resistance exercise were among the most effective modalities for improving pro-atherogenic lipid profiles, particularly LDL-C and triglycerides. According to their report, the benefits of HIIT begin to appear at around 250 MET-min/w, reach their maximum between 1000 and 1500 MET-min/w, and disappear at about 1800 MET-min/w [143].

Recent literature has also highlighted the possibility that very high intensity of endurance exercise may be associated with greater coronary plaque burden and calcification in some athletes. A 2025 review by Claessen et al. found that engaging in high-volume, long-term endurance exercise at a high intensity may be linked to coronary atherosclerosis. Their review suggested that the greatest benefit occurs at approximately 3 h per week of moderate-intensity exercise with good CRF, beyond which the protective effect may diminish [144]. On the other hand, Gomes-Neto et al. reported in a 2024 network meta-analysis that all major exercise modalities reduced mortality in patients with coronary heart disease, with HIIT showing the greatest improvement in peak oxygen consumption [145]. Similarly, Laukkanen et al. reported in a meta-analysis of 37 cohorts that each 1-MET higher level of cardiorespiratory fitness was associated with a relative risk of 0.89 (95% CI, 0.86–0.92) for all-cause mortality [12].

Potential adverse effects should also be considered when prescribing high-intensity or high-volume exercise. Although habitual physical activity reduces long-term cardiovascular risk, vigorous exertion transiently increases the risk of acute myocardial infarction and sudden cardiac death, particularly among untrained individuals and those with underlying cardiovascular disease; however, the absolute risk remains low [146]. Prolonged high-volume endurance training has also been associated with an increased risk of atrial fibrillation, particularly among middle-aged male endurance athletes [146]. Therefore, exercise intensity should be increased gradually and individualized according to baseline fitness, symptoms, comorbidities, and underlying cardiac disease.

Overall, current evidence provides pragmatic population-level targets, but further investigation is needed to determine the optimal type, volume, and intensity of exercise for different individuals, particularly across age and sex groups. High-volume or high-intensity exercise may increase atherosclerotic plaque burden in some populations, especially men, while still reducing mortality through other mechanisms, including improved CRF, risk-factor modification, and greater plaque calcification and stabilization.

## 6. Clinical Implications

The available evidence supports exercise as a core component of primary prevention of atherosclerotic CVD. For most adults, the most important step is moving from inactivity to regular activity, since the largest reduction in cardiovascular risk appears to occur at relatively modest exercise volumes [6,128,129]. A practical clinical strategy is to advise patients to achieve 1000 MET-min/w, or about 3 h of moderate to high intensity exercise. This amount should be increased gradually, considering factors like age, current fitness level, existing health conditions, and personal preferences, with a goal of good CRF [6,12,133,143,145].

For selected individuals, HIIT may provide additional improvements in CRF and some cardiometabolic parameters, although it may be best suited to those with adequate baseline fitness or supervised settings [143,145]. Resistance training should also be encouraged, ideally in combination with aerobic exercise, because it improves metabolic health, functional capacity, and overall cardiovascular profile [133,134,135].

From a mechanistic standpoint, exercise favorably affects several pathways involved in atherosclerosis, including lipid metabolism, insulin sensitivity, blood pressure, oxidative stress, inflammation, and endothelial function, as discussed above. At the same time, exercise should not be viewed as a substitute for evidence-based medical therapy in patients at elevated risk. In contemporary prevention practice, physical activity works best as part of a broader strategy that also includes control of lipids, blood pressure, diabetes, body weight, and smoking, with pharmacologic therapy when indicated [6,133,147].

An important clinical nuance is that greater plaque calcification or higher coronary artery calcium burden observed in some highly active individuals, especially endurance athletes, should not automatically be interpreted as evidence of harm [13,14,103]. Current data suggest that highly active individuals may have more plaques, while overall cardiovascular and all-cause mortality often remain lower than in less active populations [12,128,144,145]. Therefore, imaging findings should be interpreted in the context of overall risk profile, symptoms, fitness, and plaque characteristics rather than CAC score alone. Regular exercise in non-athletes has not been shown to increase plaque burden compared with sedentary individuals, and fear of calcification should not discourage routine physical activity [104,107,108].

Finally, exercise prescription should acknowledge real-world barriers and safety considerations. Long-term adherence is often limited by time constraints, musculoskeletal symptoms, socioeconomic factors, access to safe exercise environments, and low baseline fitness. In previously inactive individuals, exercise plans may need to begin with supervised or structured programs and progress gradually [132,133].

Cardiovascular prevention should also follow a life-course approach beginning in childhood and adolescence. Physical inactivity, excess adiposity, and other cardiometabolic risk factors may emerge early and persist into adulthood, increasing cumulative lifetime cardiovascular risk. Early education promoting regular physical activity, healthy nutrition, and weight management, together with age-appropriate assessment of anthropometric and cardiometabolic risk, may help establish healthier behaviors and reduce long-term ASCVD risk [148].

## 7. Future Directions

The current literature also leaves important unanswered questions regarding the optimal type, intensity, and upper safe limit of exercise across different age groups and between men and women.

Future research should focus on developing individualized exercise prescriptions over broad recommendations for the general population. Integrating clinical characteristics with genetics, metabolomics, and vascular imaging may help identify the most effective type, intensity, and duration of exercise for different population groups, while also clarifying whether very high exercise volumes carry different vascular effects in selected populations.

Wearable devices and digital health tools could support individualized exercise prescription by providing objective longitudinal measures of activity and physiological response. In an FHS sub-study, higher device-measured step counts were associated with lower estimated CVD risk, supporting their potential research and clinical utility [116].

## Figures and Tables

**Table 1:** Major clinical studies evaluating physical activity, exercise, and atherosclerotic cardiovascular disease.

Study	Population	PA	Outcome	Follow-Up	Adjustments	Results
FHS, Shortreed et al., 2013 [115]	4729 adults; prospective cohort	Self-reported habitual PA	Incident CVD and CVD mortality	40 y	Marginal structural model; time-varying confounders	Long-term PA was associated with lower CVD and all-cause mortality, although its association with incident CVD was not statistically significant
ARIC, Florido et al., 2016 [117]	9996 adults without baseline ASCVD; mean follow-up 20.9 years	Self-reported moderate-to-vigorous PA	Incident ASCVD	20.9 y	Age, sex, race, education, BMI, smoking, alcohol	Greater PA was associated with lower ASCVD risk regardless of family history of premature CHD
MESA, German et al., 2022 [122]	6777 adults without baseline clinical CVD	Self-reported PA	CAC-stratified CVD outcomes	14.4 y	Demographics, BMI, smoking, BP, diabetes, medications, lipids	Higher PA was associated with lower CVD risk among participants with CAC <100, and high activity was not associated with worse outcomes at any CAC level
PURE, Lear et al., 2017 [121]	130,843 adults from 17 countries; prospective cohort	Recreational and nonrecreational PA	Major CVD and mortality	6.9 y	Sociodemographic and CVD risk factors; study clustering	Higher PA was associated with lower mortality and major CVD across countries of different income, with benefits of both recreational and nonrecreational activity
CARDIA, Lee et al., 2009 [123]	2373 Black and White young adults; 15-year follow-up	Treadmillmeasured CRF	Incident CAC	15 y	Demographics, smoking, waist, alcohol, PA, BP, diabetes, insulin	High baseline fitness was associated with a lower likelihood of CAC 15 years later compared with low fitness
MESA, Delaney et al., 2013 [124]	5656 adults without clinical CVD	Self-reported PA	Incident CAC and progression to PAD based on ABI	variable	Demographics, conventional CVD and socioeconomic factors	Vigorous activity was associated with lower incident CAC, intentional exercise was associated with lower incident PAD, and sedentary behavior was associated with greater CAC progression
Cooper Center, DeFina et al., 2019 [127]	21,758 generally healthy men; prospective cohort	Self-reported PA	CAC and all-cause/CVD mortality	10.4 y	Age, BMI, glucose, cholesterol, SBP, smoking	Very high activity was associated with a greater prevalence of CAC but not with higher all-cause or CVD mortality, including among men with elevated CAC.
Sung et al., 2021 [125]	25,485 adults with serial CAC examinations; prospective cohort	Self-reported PA	CAC prevalence and progression	5 y	Demographic, lifestyle and cardiometabolic factors	PA showed a graded positive association with CAC prevalence and progression; the clinical significance of this finding remained uncertain.
Cooper Clinic, Shuval et al., 2024 [126]	8771 healthy adults; mean follow-up 7.8 years	Serial self-reported PA	CAC progression	7.8 y	Age/CAC, scanner, smoking, BMI, glucose, cholesterol, SBP, statin	High and very high activity volumes were not associated with the relative rate of CAC progression or progression to CAC ≥ 100
Möhlenkamp et al., 2008 [103]	108 male marathon runners aged ≥50 years with matched controls	Marathon participation and training history	CAC, myocardial fibrosis, and coronary events	21.3 m	Age- and Framingham-risk-matched controls	Marathon runners had greater CAC than expected from their conventional risk profile
MARC-1, Aengevaeren et al., 2017 [13]	284 middle-aged and older male recreational athletes	Lifelong exercise volume and intensity	CAC and CCTA-defined plaque	NA	Age, BMI, SBP, smoking, medications, cholesterol, family history	Higher lifelong exercise was associated with greater CAC and plaque prevalence; the most active participants more often had predominantly calcified plaques
Merghani et al., 2017 [14]	152 masters endurance athletes and 92 matched controls	Lifelong endurance training history	CAC and CCTA-defined coronary plaque	NA	Controls matched for age, sex and low Framingham risk	Male athletes had more coronary plaques than controls, but plaques in athletes were predominantly calcified; this difference was not observed among female athletes.
MARC-2, Aengevaeren et al., 2023 [130]	289 male athletes with repeat imaging after approximately 6 years	Exercise volume and intensity during follow-up	CAC and CCTA plaque progression	6.3 y	Baseline CAC/plaque, age and conventional CVD risk factors	Exercise volume was not associated with plaque progression; very vigorous exercise was associated with greater CAC and calcified-plaque progression, whereas vigorous exercise was associated with less progression.
Master@Heart, De Bosscher et al., 2023 [102]	558 healthy men: 191 lifelong athletes, 191 late-onset athletes, and 176 active controls	Endurance-training history and measured fitness	CCTA-defined plaque burden and composition	NA	Age, BMI, BP, lipids, HbA1c, family history	Lifelong endurance athletes had a greater overall burden of calcified, mixed, and noncalcified plaque than active controls, despite a favorable risk profile.
Papatheodorou et al., 2024 [15]	196 female endurance athletes and 59 controls	Lifelong endurance-training history	CAC and CCTA-defined plaque	NA	Low-risk controls; age, SBP and HDL-C in final model	Female endurance athletes did not have greater CCTA defined coronary plaque burden than controls and had a lower prevalence of CAC above the age-adjusted 50th percentile, suggesting potentially important sex differences.

PA: physical activity; CVD: cardiovascular disease; y: year; BMI: body mass index; ASCVD: atherosclerotic cardiovascular disease; CHD: coronary heart disease; CAC: coronary artery calcium; BP: blood pressure; ABI: ankle brachial index; PAD: peripheral artery disease; NA: not applicable; SBP: systolic blood pressure; m: month; CCTA: coronary computed tomography angiography; HDL-C: high-density lipoprotein cholesterol.

## Data Availability

No new data were created or analyzed in this study. Data sharing is not applicable to this article.
